# Austerity, economic hardship and access to medications: a repeated cross-sectional population survey study, 2013–2020

**DOI:** 10.1136/jech-2022-219706

**Published:** 2023-01-24

**Authors:** Katri Aaltonen

**Affiliations:** 1 INVEST Research Flagship Center, University of Turku, Turku, Finland; 2 Kela Research, The Social Insurance Institution of Finland, Helsinki, Finland

**Keywords:** healthcare disparities, health inequalities, health policy, social sciences

## Abstract

**Background:**

In Finland, austerity measures included an increase in medication and healthcare copayments and a decrease in many social security allowances. This study examines whether austerity coincided with an increase in socioeconomic inequality in access to medications (going short of medications because of lack of money) and whether medication access problems increased more than other forms of economic hardship (going short of food or physician visits).

**Methods:**

Pooled cross-sectional population surveys collected in 2013–2015, 2018 and 2020 (n=139 324) and multinomial logistic regression, with interaction between study year and economic activity (EA) (full-time work vs part-time work/retirement; old age retirement; unemployment; disability/illness; family; student), were used to estimate the effect of EA on the probability of experiencing economic hardship (no hardship/hardship including medication problems/hardship excluding medication problems) and how it varies across years.

**Results:**

Working-age adults outside full-time employment have a higher risk of economic hardship than full-time workers, and old age retirees have a lower risk. In 2018, when austerity was most pronounced, economic hardship including medication problems increased for the disabled/ill (women and men), unemployed (women) and part-time workers/retirees (men), significantly more than for full-time workers. Hardship excluding medication access problems either decreased or remained unchanged.

**Conclusion:**

Austerity coincided with increasing economic hardship among vulnerable groups, thus exacerbating socioeconomic inequalities. Strengthening the role for medication access problems suggests that medication copayment increases contributed to this accumulating disadvantage.

**WHAT IS ALREADY KNOWN ON THIS TOPIC**

- Healthcare coverage restrictions, including increases in patient payments, were a common feature of austerity in Europe after the 2008 financial crisis.
- In Finland, austerity was most pronounced in 2015–2019, and it included increases in health payments, including medication copayments, and decreases of social allowances.
- The effects of policies related to health payments and cash transfers have been evaluated separately in the context of each subfield; thus, the complementary and cumulative effects could be unnoticed.

**WHAT THIS STUDY ADDS**

- This study examined patterns of economic hardship related to costs for medications in relation to other necessities (food and physician visits) by economic activity.
- The study found exacerbating socioeconomic inequities during pronounced austerity, with working-age individuals with disability/illness, unemployed women and part-time working/retired men becoming even more disadvantaged in relation to their counterparts in full-time employment.
- Individuals in these groups were more likely to experience economic hardship during pronounced austerity, and economic hardship was more likely to include medication affordability problems.

**HOW THIS STUDY MIGHT AFFECT RESEARCH, PRACTICE OR POLICY**

- Disadvantage increased most among groups that are likely to have been exposed to several austerity measures simultaneously (increasing copayments and decreasing replacement rate of allowances).
- To balance sustainability issues and health policy goals, policy-makers should pay attention on the effects of various austerity policies that accumulate in vulnerable subgroups.

## Introduction

As a response to the global financial crisis affecting European economies since 2008, the European Commission, European Central Bank and International Monetary Fund unanimously promoted austerity measures. In Finland, austerity was widely applied throughout the 2010s, particularly in 2015–2019.^1^ Since then, the international institutions have been advocating for higher health and social spending, while also addressing socioeconomic inequalities, and austerity is perceived to have undermined health system resilience and progress towards universal health coverage in Europe.^2^

Healthcare and other in-kind transfers increase household economic resources.^3–5^ Consequently, generous provision of public healthcare along with social assistance programmes is linked with lower odds of material deprivation.^6^ Nevertheless, in recent decades, a common austerity policy applied in response to growing healthcare costs has been coverage restrictions, often through increased direct health payments.^7 8^ Direct health payments refer to costs that users are obliged to pay directly for healthcare goods or services at the time of use. They comprise cost sharing (copayments, user charges, deductibles, etc), self-medication (eg, over-the-counter medications), informal payments and expenditure due to unavailability of formal care.^9 10^

Coverage restrictions refer to policy changes that shift healthcare costs from third-party payers to the patients, including increases in direct payments. Restrictions may result in increasing income inequality and recommodification of healthcare; that is, making access more dependent on an individual’s ability to pay and market position.^9^ However, in the context of comprehensive welfare states, the commodification of healthcare should be examined as part of the overall social system.^5 11^ This is because increasing households’ purchasing power through taxation and cash transfers can buffer inequitable effects of direct payments.^12 13^

In previous research, unmet healthcare needs have typically been measured in relation to doctor visits or other services, disregarding that households’ health spending is often driven by outpatient medications.^10^ Furthermore, individuals respond differently to economic pressures; some may go without care, some without medications and others may adopt other coping strategies.^14^ Thus, in this study, I focus on the role of medication access problems, and these problems are examined in relation to other types of economic hardship: going short of physician visits or food because of lack of money.

Finland offers an interesting case to examine the patterns of economic hardship. In 2015–2019, austerity policies simultaneously increased patient payments for medications and healthcare and decreased the replacement rate of many cash transfers. Medication copayments increased notably, and these increases mainly affected individuals with chronic illnesses. Previous studies revealed coinciding decrease in medication consumption and increase in economic problems.^15–18^ I complement previous evidence by examining financial access to medicines as part of more general patterns of economic hardship. I ask whether austerity coincided with exacerbation of inequalities, and whether hardship related to medications developed differently than other economic problems.

## Methods

### Settings

Finland is a Nordic European country with a comprehensive social security system, including universal access to healthcare and prescription medicines. Nevertheless, patient payments disproportionately burden poorer households, comprising mostly retired, disabled or long-term unemployed.^19 20^ Multiple and overlapping coverage schemes, long waiting lists and complex and heavy copayments were identified as key factors creating inequities.^19 20^ Occupational system provides most employees and entrepreneurs with primary healthcare services, including medical care with no copayments, negligible waiting times and lower gatekeeping, whereas outsiders rely on public services, subject to long waiting times, strict gatekeeping and nearly always copayments. Consequently, those with lower socioeconomic status have a higher probability of using only public services or not using primary services at all.^21^ The incidence of catastrophic health spending, driven by prescription medicines and outpatient care, is higher than that in other Nordic countries, and unmet needs are more prevalent than in many other Western European countries.^19^

During the study period, the following health payment increases were implemented: public healthcare user charges were increased in 2015 and 2016, and copayment increase was targeted to prescription medications in 2013, 2016 and 2017, with the latter two leading to increase particularly among the chronically ill.^16–19^ Cuts on reimbursements for private healthcare and private dental care were implemented in 2015 and 2016, and reimbursements for travel costs to healthcare were reduced in 2013, 2015, 2016 and 2018.^19^

In terms of tax benefit policies, the three governments that operated during the period differed in their approaches. During Prime Minister (PM) Katainen/Stubb’s tenure (2011–2015), expansionary tax benefit policies reduced the poverty risk rate.^22^ During PM Sipilä’s tenure (2015–2019), poverty outcomes slightly worsened.^23^ The poverty impact of tax benefit policies implemented by PM Rinne/Marin (2019-ongoing) is yet to be examined; however, in 2020, the government slightly increased basic social security allowances and abolished a sanction model for the unemployed.^24^ Moreover, 2020 was characterised by the COVID19-pandemic. For example, temporary mechanisms (unemployment benefits, loans, funding and cost support) were implemented, to sustain the livelihood of entrepreneurs and the self-employed.^25 26^

### Study design, population and data sources

Nationally representative repeated cross-sectional population surveys were conducted by the Finnish Institute for Health and Welfare to monitor the health, well-being and service use of population groups in Finland, nationally and regionally.^27 28^ In 2013, 2014 and 2015, large national random samples (n=49 865, 19 576 and 20 338, respectively) were collected in the Regional Health and Well-being study (ATH) and in 2018 (n=26 422) and 2020 (n=28 199), in the National FinSote study. ATH and FinSote used a similar methodology, and the latter survey contained some of the prior survey questions.

### Outcomes

Economic hardship was measured using the following three questions: ‘Have you within the past 12 months ever: (i) feared that you will run out of food before you can get money to buy more? (ii) been unable to buy medicines because you did not have any money? (iii) not visited a doctor because you did not have any money?’ The response options for each question (i*–*iii) were ‘(a) no’ and ‘(b) yes’. Observations with missing data for all three questions were excluded.

To examine the role of medication access problems in relation to other types of hardship, an outcome variable with three mutually exclusive categories was composed: 0=no hardship, 1=hardship including medication access problems and 2=hardship excluding medication access problems.

### Independent variables

The main independent variables of interest were economic activity (EA) and the study year. EA was used to define individual’s market position, thus distinguishing population subgroups by their reliance on redistributive welfare state policies. In the Finnish context, labour market attachment also determines access to occupational healthcare services. All analyses were stratified by sex, because of the known differences in healthcare needs and use patterns.

To determine EA, the following question was used: ‘Are you currently mainly: (a) in full-time work; (b) in part-time work or part-time retired (hereafter: part-time work/retirement); (c) retired due to age (hereafter: old age retirement); (d) on disability pension or receiving rehabilitation benefit (hereafter: disability/illness); (e) unemployed or laid off (hereafter: unemployed); (f) taking care of children at home (hereafter: family); (g) student; (h) something else, what? (with free text field). Free text answers were assigned to classes a–g when possible. Individuals on sickness leave/allowance were classified under (d) because sickness allowance can be paid in case of short-term disability for up to 300 days, and it also applies to the unemployed. Individuals aged 69 years or older were classified as old age retirees (c).

### Data analysis

First, a multinomial logistic regression model was fitted by regressing financial hardship (no hardship/hardship including medication access problems/hardship excluding medication access problems) on EA categories and the study year. Because austerity was most pronounced in 2018, it was used as the reference category.

Second, to test the interactive effect of EA and study year, the product term (EA×year) was added to the model. The average marginal effects (AMEs) of EA (reference: full-time work) were calculated for each year. To formally test whether the year modifies the effect of EA, the difference in AMEs between 2018 and other years was calculated, with a test of differences, which also serves as a test for interaction.^29^ AMEs are interpreted as percentage point (ppt) differences in probabilities in relation to the reference group. Unlike ORs, AMEs allow comparison of effect sizes across groups and models.^30^

The study population included all individuals with no missing data on the variables used in the analyses. Respondents whose EA did not fall into the afore-mentioned groups a–g were excluded. Men in class f (family) had very few observations per year; thus, they were also excluded.

All analyses were conducted using Stata V.16.0 statistical software (Stata Corporation, College Station, Texas, USA), using packages estout^31^ and spost13_ado.^32^ Data were accessed through the Finnish Social and Health Data Permit Authority Findata remote access system, Kapseli. Weights (sampling probability, area, age, sex, marital status, education, language) and strata variables were used in all analyses to restore population representation and account for the complex sampling design.^27^ In Stata, these variables were identified using the ‘svyset’ command.

## Results

### Characteristics

The study population consisted of 139 324 individuals; therefore, 5076 (3.5% of the total 144 400) responses were excluded. Table 1 presents the characteristics of the study population.

**Table 1:** Characteristics of the study population (n, unadjusted, % of study population)

	2013	2014	2015	2018	2020	Pooled
Full dataset	49 865	19 576	20 338	26 422	28 199	144 400
Excluded (%*)	1708 (3.4%)	707 (3.6%)	900 (4.4%)	788 (3.0%)	973 (3.5%)	5076 (3.5%)
Included (study population)	48 157	18 869	19 438	25 634	27 226	139 324
Hardship, going short of medications	4102 (8.5%)	1619 (8.6%)	1715 (8.8%)	2318 (9.0%)	1804 (6.6%)	11 558 (8.3%)
Hardship, going short of physician visits	4930 (10.2%)	1960 (10.4%)	2064 (10.6%)	2509 (9.8%)	2278 (8.4%)	13 741 (9.9%)
Hardship, going short of food	3854 (8.0%)	1601 (8.5%)	1573 (8.1%)	1838 (7.2%)	1659(6.1%)	10 525(7.6%)
Hardship, at least one type	7859 (16.3%)	3182 (16.9%)	3314 (17.1%)	3831 (14.9%)	3437 (12.6%)	21 623 (15.5%)
Sex, women	27 611 (57.3%)	10 831 (57.4%)	11 092 (57.1%)	14 495 (56.6%)	15 297 (56.2%)	79 326 (56.9%)
Sex, men	20 546 (42.7%)	8038 (42.6%)	8346 (42.9%)	11 139 (43.5%)	11 929 (43.8%)	59 998 (43.1%)
Age, 20–44 years	12 559 (26.1%)	4686 (24.8%)	4888 (25.1%)	4201 (16.4%)	4791 (17.6%)	31 125 (22.3%)
Age, 45–64 years	16 383 (34.0%)	6241 (33.1%)	6363 (32.7%)	6844 (26.7%)	7146 (26.3%)	42 977 (30.9%)
Age, 65+ years	19 215 (39.9%)	7942 (42.1%)	8187 (42.1%)	14 589 (56.9%)	15 289 (56.2%)	65 222 (46.8%)
Main activity, full-time work	18 087 (37.6%)	6823 (36.2%)	6959 (35.8%)	6495 (25.3%)	7517 (27.6%)	45 881 (32.9%)
Main activity, part-time work/retirement	2000 (4.2%)	827 (4.4%)	801 (4.1%)	812 (3.2%)	899 (3.3%)	5339 (3.8%)
Main activity, old age retirement	20 933 (43.5%)	8569 (45.4%)	8787 (45.2%)	15 478 (60.4%)	15 918 (58.5%)	69 685 (50.0%)
Main activity, disability/illness	2164 (4.5%)	716 (3.8%)	797 (4.1%)	868 (3.4%)	823 (3.0%)	5368 (3.9%)
Main activity, unemployed	2021 (4.2%)	822 (4.4%)	920 (4.7%)	947 (3.7%)	999 (3.7%)	5709 (4.1%)
Main activity, family†	962 (2.0%)	381 (2.0%)	346 (1.8%)	293 (1.1%)	251 (0.9%)	2233 (1.6%)
Main activity, student	1990 (4.1%)	731 (3.9%)	828 (4.3%)	741 (2.9%)	819 (3.0%)	5109 (3.7%)

*Per cent of full dataset.

†This category was excluded from the analysis of men owing to the low number of observations; thus, the numbers in the table include only women.

The estimated proportion of individuals with at least one type of economic hardship was 20%–23% among women and 17%–18% among men (table 2). Overall, 8%–12% of the respondents reported only one type of hardship, meaning approximately half of those who reported any hardship, and 3%–5% reported all three types of hardships.

**Table 2:** Estimated proportions (with 95% CI) of respondents reporting economic hardship, by type of hardship (going short of medications, going short of physician visits, going short of food) and overlap between different types of hardship

	Women (n=79 326)					Men (n=59 998)				
	2013	2014	2015	2018	2020	2013	2014	2015	2018	2020
No hardship	0.784	0.772	0.766	0.769	0.804	0.822	0.816	0.825	0.832	0.833
	(0.778 to 0.789)	(0.763 to 0.781)	(0.757 to 0.775)	(0.755 to 0.782)	(0.794 to 0.813)	(0.816 to 0.828)	(0.805 to 0.826)	(0.815 to 0.835)	(0.816 to 0.846)	(0.822 to 0.845)
At least one type (medication and/or physician, and/or food)	0.216	0.228	0.234	0.231	0.197	0.178	0.184	0.175	0.169	0.167
	(0.211 to 0.222)	(0.219 to 0.237)	(0.225 to 0.243)	(0.218 to 0.245)	(0.187 to 0.206)	(0.172 to 0.184)	(0.174 to 0.195)	(0.166 to 0.185)	(0.154 to 0.184)	(0.156 to 0.178)
Total proportions of respondents with different types of hardship without considering overlap										
Going short of medications (with or without other types)	0.108	0.119	0.120	0.130	0.101	0.096	0.088	0.088	0.102	0.087
	(0.104 to 0.113)	(0.112 to 0.126)	(0.113 to 0.127)	(0.120 to 0.142)	(0.094 to 0.109)	(0.091 to 0.101)	(0.080 to 0.096)	(0.081 to 0.096)	(0.091 to 0.115)	(0.079 to 0.096)
Going short of physician visits (with or without other types)	0.138	0.144	0.149	0.160	0.131	0.103	0.103	0.098	0.101	0.103
	(0.134 to 0.143)	(0.137 to 0.152)	(0.142 to 0.157)	(0.147 to 0.172)	(0.123 to 0.139)	(0.098 to 0.108)	(0.095 to 0.111)	(0.091 to 0.106)	(0.090 to 0.113)	(0.094 to 0.113)
Going short of food (with or without other types)	0.114	0.123	0.117	0.118	0.104	0.101	0.107	0.100	0.098	0.099
	(0.109 to 0.118)	(0.116 to 0.131)	(0.110 to 0.124)	(0.107 to 0.130)	(0.097 to 0.112)	(0.096 to 0.106)	(0.098 to 0.116)	(0.092 to 0.109)	(0.086 to 0.110)	(0.090 to 0.109)
All combinations of hardship types and their overlap										
No hardship	0.784	0.772	0.766	0.769	0.804	0.822	0.816	0.825	0.832	0.833
	(0.778 to 0.789)	(0.763 to 0.781)	(0.757 to 0.775)	(0.755 to 0.782)	(0.794 to 0.813)	(0.816 to 0.828)	(0.805 to 0.826)	(0.815 to 0.835)	(0.816 to 0.846)	(0.822 to 0.845)
Food only (not medications, not physician)	0.038	0.038	0.039	0.029	0.030	0.038	0.044	0.041	0.023	0.034
	(0.036 to 0.041)	(0.034 to 0.042)	(0.035 to 0.044)	(0.024 to 0.035)	(0.026 to 0.035)	(0.034 to 0.041)	(0.038 to 0.050)	(0.035 to 0.046)	(0.018 to 0.029)	(0.028 to 0.040)
Physician only (not medications, not food)	0.055	0.054	0.060	0.053	0.050	0.034	0.039	0.035	0.031	0.034
	(0.052 to 0.058)	(0.050 to 0.059)	(0.055 to 0.065)	(0.047 to 0.061)	(0.045 to 0.055)	(0.031 to 0.037)	(0.034 to 0.044)	(0.031 to 0.040)	(0.025 to 0.038)	(0.029 to 0.040)
Medications only (not food, not physician)	0.021	0.023	0.025	0.026	0.019	0.021	0.021	0.018	0.021	0.017
	(0.019 to 0.023)	(0.020 to 0.026)	(0.022 to 0.028)	(0.022 to 0.031)	(0.016 to 0.022)	(0.019 to 0.024)	(0.018 to 0.025)	(0.015 to 0.021)	(0.017 to 0.027)	(0.013 to 0.021)
Food and physician (not medications)	0.014	0.017	0.015	0.019	0.016	0.011	0.014	0.011	0.013	0.012
	(0.013 to 0.016)	(0.014 to 0.020)	(0.013 to 0.018)	(0.014 to 0.025)	(0.013 to 0.020)	(0.009 to 0.013)	(0.011 to 0.018)	(0.008 to 0.014)	(0.009 to 0.018)	(0.009 to 0.016)
Food and medications (not physician)	0.018	0.023	0.021	0.017	0.017	0.016	0.016	0.019	0.023	0.013
	(0.017 to 0.020)	(0.020 to 0.027)	(0.018 to 0.025)	(0.014 to 0.021)	(0.014 to 0.021)	(0.014 to 0.019)	(0.013 to 0.020)	(0.015 to 0.023)	(0.017 to 0.032)	(0.010 to 0.017)
Physician and medications (not food)	0.027	0.027	0.033	0.034	0.024	0.022	0.018	0.022	0.019	0.017
	(0.025 to 0.029)	(0.024 to 0.031)	(0.029 to 0.037)	(0.028 to 0.041)	(0.021 to 0.028)	(0.020 to 0.025)	(0.015 to 0.021)	(0.019 to 0.026)	(0.015 to 0.024)	(0.014 to 0.021)
All three types of hardship (food and medication and physician)	0.042	0.046	0.041	0.054	0.041	0.037	0.033	0.030	0.039	0.041
	(0.040 to 0.045)	(0.041 to 0.051)	(0.037 to 0.046)	(0.046 to 0.062)	(0.036 to 0.047)	(0.033 to 0.040)	(0.028 to 0.038)	(0.026 to 0.035)	(0.032 to 0.046)	(0.035 to 0.047)

### Main effects of EA and year

Table 3 first presents the probabilities of reporting no hardship, hardship including medication problems or hardship excluding medication problems, by EA in relation to full-time workers, net of study year. Among both women and men, old age retirees had the lowest probability of reporting any hardship. Retired women had 9ppts and men had four ppts higher probability of reporting no hardship than full-time workers. Old age retirees had a lower probability of reporting hardship, including and excluding medication access problems.

**Table 3:** Average marginal effects (AMEs, with SE) of the independent variables on the probability of reporting economic hardship including or excluding medication access problems, by sex

	Women (n=79 326)						Men (n=59 998)					
	No hardship		Hardship including medications		Hardship excluding medications		No hardship		Hardship including medications		Hardship excluding medications	
	AME (SE)	P value	AME (SE)	P value	AME (SE)	P value	AME (SE)	P value	AME (SE)	P value	AME (SE)	P value
Economic activity (ref=full-time work)												
Part-time work/retirement	−**0.108** (**0.010**)	**<0.001**	**0.062** (**0.008**)	**<0.001**	**0.046** (**0.008**)	**<0.001**	−**0.157** (**0.018**)	**<0.001**	**0.119** (**0.016**)	**<0.001**	**0.038** (**0.011**)	**0.001**
Old age retired	**0.088** (**0.004**)	**<0.001**	−**0.032** (**0.003**)	**<0.001**	−**0.056** (**0.003**)	**<0.001**	**0.042** (**0.004**)	**<0.001**	−**0.008** (**0.003**)	**0.005**	−**0.035** (**0.003**)	**<0.001**
Disability/illness	−**0.290** (**0.012**)	**<0.001**	**0.245** (**0.012**)	**<0.001**	**0.045** (**0.008**)	**<0.001**	−**0.266** (**0.013**)	**<0.001**	**0.237** (**0.013**)	**<0.001**	**0.028** (**0.007**)	**<0.001**
Unemployed	−**0.274** (**0.012**)	**<0.001**	**0.174** (**0.011**)	**<0.001**	**0.100** (**0.010**)	**<0.001**	−**0.287** (**0.012**)	**<0.001**	**0.164** (**0.010**)	**<0.001**	**0.123** (**0.010**)	**<0.001**
Family	−**0.047** (**0.012**)	**<0.001**	**0.030** (**0.010**)	**0.002**	**0.017** (**0.009**)	**0.049**						
Student	−**0.180** (**0.011**)	**<0.001**	**0.101** (**0.009**)	**<0.001**	**0.079** (**0.009**)	**<0.001**	−**0.175** (**0.014**)	**<0.001**	**0.086** (**0.011**)	**<0.001**	**0.089** (**0.010**)	**<0.001**
Year (ref=2018)												
2013	**0.016** (**0.007**)	**0.027**	−**0.022** (**0.006**)	**<0.001**	0.006 (0.006)	0.242	−0.006 (0.008)	0.428	−0.009 (0.006)	0.165	**0.015** (**0.005**)	**0.006**
2014	0.003 (0.008)	0.683	−0.011 (0.006)	0.083	0.008 (0.006)	0.191	−0.015 (0.009)	0.087	−**0.015** (**0.007**)	**0.033**	**0.030** (**0.006**)	**<0.001**
2015	<0.001 (0.008)	0.996	−0.012 (0.006)	0.054	**0.012** (**0.006**)	**0.042**	−0.003 (0.009)	0.693	−**0.015** (**0.007**)	**0.025**	**0.019** (**0.006**)	**0.003**
2020	**0.034** (**0.008**)	**<0.001**	−**0.030** (**0.007**)	**<0.001**	−0.005 (0.006)	0.436	0.004 (0.009)	0.669	−**0.016** (**0.007**)	**0.028**	0.012 (0.007)	0.067

Results are based on multinomial logistic regression (main effects).

Bold values denote statistical significance at the p < 0.05 level.

Conversely, all groups with working-age individuals partly or entirely outside full-time employment had a higher probability of reporting hardship than their counterparts in full-time work. In terms of medication access problems, individuals with disability/illness were particularly disadvantaged: they had 24–25 ppts higher probability of reporting such hardship, and 3–5 ppts higher probability of reporting other types of hardship. The unemployed had a relatively high probability of reporting any hardship; however, their experiences were less skewed towards hardship including medication access problems. Compared with full-time workers, they had 16–17 ppts higher probability of reporting hardship including medication access problems, and 10–12 ppts higher probability of reporting other types of hardship.

Second, table 3 presents the probabilities of reporting hardship by year in relation to 2018, net of EA. Across years, hardship including medication access problems tended to be slightly more common in 2018 than in other years, and other types of hardship were slightly less common. However, the effect sizes were small (≤3 ppts), and the differences were not always statistically significant. The prevalence of any hardship remained relatively stable over the years.

### Patterns of economic hardship by EA

Next, interactive effects were examined, that is, whether patterns of economic hardship developed differently over time across EA groups. The predicted probabilities of respondents reporting no hardship, or hardship including or excluding medication access problems, by EA and year are presented online supplemental tables S1 and S2. The AMEs of EA represent the differences between full-time workers (reference groups) and the other groups by year. For example, in 2018, the estimated probability for women working full-time to report no hardship was 81%, and for unemployed women, the probability was 47%. The difference in the probability (ie, AME) between these groups in 2018 was 34 ppts (p<0.001), indicating that unemployed women had 34 ppts higher probability of reporting hardship than women working full time. In 2013–2015, the difference between these groups was smaller (23–25 ppts).

10.1136/jech-2022-219706.supp1 Supplementary data

To further test whether the difference in the AME of EA varied statistically significantly between year 2018 and the other years (ie, whether the interaction is statistically significant), tables 4–5 present the differences in AMEs of EA between 2018 and the other years. To continue the preceding example, table 4 shows that the difference in the probability of reporting no hardship between unemployed and full-time working women is 9–11 ppts larger in 2018 than in 2013–2015 (p<0.05). This means that in 2018, in terms of economic hardship, unemployed women were even more disadvantaged in relation to full-time workers than in 2013–2015.

**Table 4:** Difference in the average marginal effect (AME with SE) of economic activity in 2013, 2014, 2015 and 2020 vs 2018, women

	Compared years	No hardship			Hardship including medications			Hardship excluding medications		
		AME difference	SE	P value	AME difference	SE	P value	AME difference	SE	P value
Economic activity (ref: full-time work)										
Part-time work/retirement	2018 vs 2013	−0.061	0.038	0.108	0.043	0.031	0.172	0.018	0.030	0.539
	2018 vs 2014	−0.045	0.042	0.286	0.029	0.034	0.393	0.015	0.033	0.645
	2018 vs 2015	−0.029	0.042	0.490	0.013	0.034	0.697	0.015	0.033	0.638
	2018 vs 2020	−0.075	0.043	0.079	0.030	0.035	0.388	0.045	0.033	0.177
Old age retirement	2018 vs 2013	−0.016	0.014	0.245	0.019	0.010	0.062	−0.003	0.011	0.803
	2018 vs 2014	−0.030	0.015	0.052	**0.029**	**0.011**	**0.010**	0.001	0.012	0.934
	2018 vs 2015	−0.009	0.015	0.573	0.016	0.011	0.169	−0.007	0.012	0.559
	2018 vs 2020	−0.007	0.016	0.648	0.012	0.011	0.275	−0.005	0.012	0.670
Disability/Illness	2018 vs 2013	−**0.125**	**0.042**	**0.003**	**0.133**	**0.044**	**0.003**	−0.008	0.028	0.767
	2018 vs 2014	−**0.163**	**0.047**	**0.001**	**0.165**	**0.049**	**0.001**	−0.002	0.032	0.962
	2018 vs 2015	−0.075	0.047	0.109	**0.120**	**0.048**	**0.013**	−0.046	0.033	0.167
	2018 vs 2020	−0.042	0.050	0.396	0.049	0.053	0.353	−0.006	0.033	0.848
Unemployment	2018 vs 2013	−**0.087**	**0.043**	**0.044**	**0.122**	**0.042**	**0.004**	−0.034	0.031	0.265
	2018 vs 2014	−**0.106**	**0.047**	**0.025**	**0.124**	**0.045**	**0.006**	−0.018	0.035	0.614
	2018 vs 2015	−**0.095**	**0.047**	**0.043**	**0.107**	**0.045**	**0.018**	−0.012	0.034	0.725
	2018 vs 2020	−0.047	0.049	0.335	**0.114**	**0.046**	**0.014**	−0.067	0.037	0.068
Family	2018 vs 2013	−0.036	0.047	0.449	0.041	0.040	0.308	−0.005	0.033	0.873
	2018 vs 2014	−0.050	0.051	0.328	0.035	0.043	0.412	0.014	0.035	0.685
	2018 vs 2015	−0.011	0.052	0.829	**0.086**	**0.042**	**0.040**	−0.075	0.038	0.051
	2018 vs 2020	−0.037	0.055	0.503	0.046	0.046	0.321	−0.009	0.038	0.809
Student	2018 vs 2013	0.009	0.038	0.814	−0.023	0.030	0.446	0.014	0.031	0.658
	2018 vs 2014	−0.008	0.043	0.855	−0.029	0.034	0.404	0.036	0.035	0.293
	2018 vs 2015	−0.016	0.042	0.713	−0.011	0.033	0.742	0.027	0.034	0.439
	2018 vs 2020	−0.072	0.044	0.102	0.015	0.035	0.672	0.057	0.035	0.101

Results are based on multinomial logistic regression, with interaction (year×economic activity).

Bold values denote statistical significance at the p < 0.05 level.

**Table 5:** Difference in the average marginal effect (AME with SE) of economic activity in 2013, 2014, 2015 and 2020 vs 2018, men

	Compared years	No hardship			Hardship including medications			Hardship Excluding medications		
		AME difference	SE	P value	AME difference	SE	P value	AME difference	SE	P value
Economic activity (ref: full-time work)										
Part-time work/retirement	2018 vs 2013	−**0.152**	**0.065**	**0.020**	**0.154**	**0.064**	**0.017**	−0.002	0.030	0.952
	2018 vs 2014	−**0.165**	**0.070**	**0.019**	**0.181**	**0.066**	**0.006**	−0.016	0.037	0.661
	2018 vs 2015	−0.136	0.072	0.058	**0.154**	**0.068**	**0.024**	−0.018	0.037	0.624
	2018 vs 2020	−0.060	0.074	0.418	0.078	0.071	0.270	−0.018	0.041	0.662
Old age retirement	2018 vs 2013	−0.019	0.012	0.129	**0.018**	**0.009**	**0.044**	0.001	0.009	0.939
	2018 vs 2014	−**0.038**	**0.014**	**0.008**	**0.023**	**0.010**	**0.026**	0.015	0.011	0.155
	2018 vs 2015	−0.014	0.014	0.320	0.010	0.010	0.336	0.004	0.010	0.685
	2018 vs 2020	−0.007	0.014	0.617	0.011	0.010	0.281	−0.004	0.011	0.724
Disability/Illness	2018 vs 2013	−**0.119**	**0.047**	**0.011**	**0.149**	**0.046**	**0.001**	−0.030	0.018	0.099
	2018 vs 2014	−**0.112**	**0.053**	**0.034**	**0.190**	**0.050**	**<0.001**	−**0.078**	**0.027**	**0.005**
	2018 vs 2015	−0.102	0.052	0.050	**0.137**	**0.050**	**0.007**	−0.035	0.024	0.145
	2018 vs 2020	−0.023	0.056	0.681	0.045	0.056	0.418	−0.022	0.023	0.336
Unemployment	2018 vs 2013	−0.004	0.042	0.933	0.005	0.033	0.879	−0.001	0.038	0.971
	2018 vs 2014	−0.004	0.048	0.935	0.015	0.038	0.682	−0.012	0.043	0.787
	2018 vs 2015	−0.034	0.046	0.470	0.035	0.036	0.330	−0.001	0.042	0.974
	2018 vs 2020	−0.043	0.049	0.376	0.040	0.038	0.292	0.003	0.043	0.937
Student	2018 vs 2013	<0.001	0.050	0.998	0.029	0.044	0.511	−0.029	0.033	0.389
	2018 vs 2014	0.006	0.056	0.907	0.029	0.047	0.542	−0.035	0.039	0.369
	2018 vs 2015	−0.031	0.053	0.555	0.053	0.045	0.241	−0.022	0.037	0.554
	2018 vs 2020	0.017	0.057	0.771	0.040	0.048	0.409	−0.056	0.040	0.164

Results are based on multinomial logistic regression, with interaction (year×economic activity).

Bold values denote statistical significance at the p < 0.05 level.

Widening gaps in hardship including medication access problems were also observed for women and men with disability/illness (12–19 ppts in 2013–2015, in comparison to 2018), and men working/retired part-time (15–18 ppts, in 2013–2015, in comparison to 2018). In these groups, the gap in the probability to experience hardship including medication access problems mostly widened, whereas the gap in the probability of experiencing other types of hardship remained relatively stable or even narrowed slightly. This indicates that the role of medication access problems strengthened.

## Discussion

This study examined patterns of economic hardship by EA over time in Finland using five cross-sectional, nationally representative population surveys. The focus was on medication access problems and their interplay with other types of hardship. The year 2018 represented pronounced austerity, to which other years were compared. The study found that economic hardship including medication access problems tended to increase in 2018; however, overall differences between years were small. In specific groups, marked increases were observed. Among the unemployed, disabled/ill and part-time workers/retirees, who were disadvantaged in relation to full-time workers throughout the period, pronounced austerity coincided with increasing inequality. In 2018, hardship in these groups included medication access problems more frequently than in other years; thus, the role of medication access problems in hardship strengthened.

Healthcare coverage restrictions during economic downturns are perceived harmful because they increase unmet needs and financial hardship, particularly among low-income households, exacerbating socioeconomic inequities in access and undermining financial protection.^2^ The Finnish case seems to have followed this logic. Vulnerable groups are likely to have been simultaneously affected by multiple austerity measures because of their low income and high healthcare needs. The buffering effects of cash benefits are likely to have decreased at the same time as the recommodification of healthcare and, in particular, medications. In 2017–2019, index cuts and freezes decreased replacement rates of many (minimum) social allowances. Unemployment benefits were further targeted by a sanction model experiment in 2018–2019, which led to notable decreases in the benefits for one-third of the unemployed, particularly the long-term unemployed and those near retirement age.^33^

Older age and longer unemployment duration are also associated with increasing healthcare needs.^34^ In the Finnish system, individuals outside full-time employment due to health reasons are likely to be spread across several EA groups used in this study. Disability pension rejections are common, after which individuals are likely to stay on low income in the long term while transitioning between the states of sickness allowance, unemployment, short-term employment and temporary rehabilitation benefits.^35 36^

Nevertheless, unemployed men seemed less sensitive to annual variations in economic hardship than unemployed women. However, overall unemployment was associated with an even higher risk of economic hardship among men than women. Women may have been more sensitive to health payment increases because of their higher service use and need. Based on previous evidence from Finland, unemployed women were more likely than unemployed men to use services overall, and they had different service use profiles. Women used primary healthcare physician and nurse services more commonly, as well as physiotherapist services, whereas men were more likely to use social services, mental health services and services related to substance abuse.^37^ These differences may lead to differential exposure to patient charges, as municipalities are required to provide specific services for free. The use of prescription medication is also generally more prevalent among women.^38^

Of note, the probability of economic hardship tended to remain stable or even slightly decrease in 2020 in many groups, despite the COVID19-pandemic. This may be because of, for example, lower consumption possibilities and targeted policy measures.^25 26^ These results are also in line with microsimulation studies, which suggested that poverty effects of the pandemic were modest in 2020.^26^

Equal access to healthcare is an important aspect of the right to health.^39^ Further accumulation of disadvantages occurs if those with lower socioeconomic position also lack access to healthcare and necessary medications. This is increasingly important in the future, as the European health systems face growing pressures of fiscal sustainability arising from ageing populations and technological innovation further exacerbated by the global pandemic and the economic consequences of the Russia–Ukraine war.^2 40^

A few caveats need to be noted. First, the survey data represented the community-dwelling population; thus, institutionalised individuals who often have high health needs were not included. Second, despite the large sample size, the specific groups examined were relatively small, and weighting might not fully account for attrition. Third, the data were cross-sectional; thus, interpretations of causality between austerity measures and economic hardship remain speculative.

## Conclusions

Based on the results, austerity coincided with an increasing gap in the probability of economic hardship between full-time workers and individuals with disability/illness, unemployed women and part-time working/retired men. In these groups, the role of medication access problems in economic hardship also strengthened, suggesting that copayment increases contributed to the accumulation of disadvantages. Sensitive groups are likely to have been affected by several austerity measures simultaneously because many depend on (minimum) social transfers for income and have high healthcare needs. In terms of the policy objectives of increasing employment rates, decreasing health inequalities and ultimately increasing equality of opportunities, the implemented policies seem contradictory. Inequitable access to healthcare and necessary medications may hamper the pursuit of all possibilities in life, including employment.

## Data Availability

Data may be obtained from a third party and are not publicly available. The author has no permission to share data; however, data access can be applied for from the centralised data permit authority Findata (https://www.findata.fi/en/).
