# Supplementary material for: Austerity, economic hardship and access to medications: a repeated cross-sectional population survey study, 2013–2020
Source: J Epidemiol Community Health. 2023 Jan 24;77(3):160–7. doi: 10.1136/jech-2022-219706 (PMC9933171; doi:10.1136/jech-2022-219706)
Supplement: Supplementary data [file jech-2022-219706supp001.pdf]

BMJ Publishing Group Limited (BMJ) disclaims all liability and responsibility arising from any reliance placed on this supplemental material which has been supplied by the author(s)

Supplementary Table S1. Predicted probabilities (95% Confidence intervals [CI]) of reporting economic hardship including or excluding medication access problems, by year and economic activity (EA), in females. Average marginal effect (AME, with standard error, se) of EA by year.

		No hardship			Hardship incl. medications			Hardship excl. medications		
**FEMALE (n=79,326)**		**Probability [95% CI]**	**AME (se)**	**p**	**Probability [95% CI]**	**AME (se)**	**p**	**Probability [95% CI]**	**AME (se)**	**p**
Full time work	2013	0.801 [0.792;0.809]			0.091 [0.085;0.097]			0.108 [0.102;0.115]		
	2014	0.780 [0.766;0.794]			0.106 [0.095;0.116]			0.114 [0.103;0.125]		
	2015	0.790 [0.776;0.804]	REF		0.100 [0.089;0.110]	REF		0.110 [0.100;0.121]	REF	
	2018	0.805 [0.782;0.827]			0.093 [0.077;0.109]			0.103 [0.085;0.121]		
	2020	0.821 [0.805;0.836]			0.080 [0.069;0.091]			0.099 [0.087;0.111]		
Part time work/retirement	2013	0.708 [0.683;0.733]	**-0.093 (0.013)**	**<0.001**	0.137 [0.117;0.156]	**0.046 (0.010)**	**<0.001**	0.155 [0.135;0.176]	**0.047 (0.011)**	**<0.001**
	2014	0.671 [0.630;0.711]	**-0.109 (0.022)**	**<0.001**	0.165 [0.132;0.197]	**0.059 (0.017)**	**0.001**	0.165 [0.133;0.197]	**0.050 (0.017)**	**0.004**
	2015	0.665 [0.625;0.704]	**-0.125 (0.021)**	**<0.001**	0.175 [0.143;0.207]	**0.075 (0.017)**	**<0.001**	0.160 [0.130;0.191]	**0.050 (0.017)**	**0.003**
	2018	0.651 [0.584;0.717]	**-0.154 (0.036)**	**<0.001**	0.181 [0.125;0.237]	**0.088 (0.030)**	**0.003**	0.168 [0.117;0.220]	**0.066 (0.028)**	**0.019**
	2020	0.742 [0.698;0.787]	**-0.079 (0.024)**	**0.001**	0.138 [0.103;0.174]	**0.058 (0.019)**	**0.002**	0.119 [0.086;0.153]	0.020 (0.018)	0.265
Old age retired	2013	0.893 [0.887;0.898]	**0.092 (0.005)**	**<0.001**	0.055 [0.051;0.060]	**-0.036 (0.004)**	**<0.001**	0.052 [0.048;0.057]	**-0.056 (0.004)**	**<0.001**
	2014	0.886 [0.876;0.896]	**0.106 (0.009)**	**<0.001**	0.059 [0.052;0.066]	**-0.046 (0.007)**	**<0.001**	0.055 [0.048;0.062]	**-0.060 (0.007)**	**<0.001**
	2015	0.875 [0.865;0.885]	**0.085 (0.009)**	**<0.001**	0.067 [0.059;0.075]	**-0.033 (0.007)**	**<0.001**	0.058 [0.051;0.065]	**-0.052 (0.007)**	**<0.001**
	2018	0.880 [0.870;0.891]	**0.076 (0.013)**	**<0.001**	0.075 [0.067;0.084]	-0.017 (0.009)	0.063	0.044 [0.037;0.052]	**-0.059 (0.010)**	**<0.001**
	2020	0.904 [0.896;0.912]	**0.083 (0.009)**	**<0.001**	0.051 [0.045;0.057]	**-0.029 (0.006)**	**<0.001**	0.045 [0.040;0.051]	**-0.054 (0.007)**	**<0.001**
Disability/ illness	2013	0.548 [0.517;0.579]	**-0.253 (0.016)**	**<0.001**	0.301 [0.272;0.329]	**0.210 (0.015)**	**<0.001**	0.151 [0.128;0.174]	**0.043 (0.012)**	**<0.001**
	2014	0.566 [0.515;0.617]	**-0.214 (0.027)**	**<0.001**	0.284 [0.236;0.331]	**0.178 (0.025)**	**<0.001**	0.150 [0.113;0.187]	0.036 (0.020)	0.068
	2015	0.488 [0.439;0.537]	**-0.302 (0.026)**	**<0.001**	0.322 [0.276;0.369]	**0.222 (0.024)**	**<0.001**	0.190 [0.151;0.229]	**0.080 (0.021)**	**<0.001**
	2018	0.427 [0.355;0.500]	**-0.377 (0.039)**	**<0.001**	0.435 [0.355;0.516]	**0.343 (0.042)**	**<0.001**	0.137 [0.090;0.184]	0.034 (0.026)	0.182
	2020	0.486 [0.426;0.546]	**-0.335 (0.032)**	**<0.001**	0.374 [0.313;0.436]	**0.294 (0.032)**	**<0.001**	0.140 [0.101;0.179]	0.041 (0.021)	0.051
Unemployed	2013	0.549 [0.517;0.581]	**-0.252 (0.017)**	**<0.001**	0.238 [0.209;0.266]	**0.147 (0.015)**	**<0.001**	0.213 [0.186;0.241]	**0.105 (0.014)**	**<0.001**
	2014	0.548 [0.499;0.597]	**-0.232 (0.026)**	**<0.001**	0.249 [0.206;0.293]	**0.144 (0.023)**	**<0.001**	0.203 [0.162;0.244]	**0.088 (0.022)**	**<0.001**
	2015	0.546 [0.499;0.593]	**-0.244 (0.025)**	**<0.001**	0.261 [0.220;0.303]	**0.161 (0.022)**	**<0.001**	0.193 [0.154;0.231]	**0.083 (0.020)**	**<0.001**
	2018	0.466 [0.392;0.540]	**-0.339 (0.040)**	**<0.001**	0.361 [0.286;0.436]	**0.268 (0.039)**	**<0.001**	0.173 [0.123;0.224]	**0.071 (0.027)**	**0.010**
	2020	0.529 [0.476;0.583]	**-0.292 (0.028)**	**<0.001**	0.234 [0.188;0.281]	**0.154 (0.024)**	**<0.001**	0.237 [0.190;0.283]	**0.138 (0.024)**	**<0.001**
Family	2013	0.761 [0.733;0.789]	**-0.039 (0.015)**	**0.009**	0.120 [0.098;0.141]	**0.029 (0.011)**	**0.011**	0.119 [0.097;0.141]	0.011 (0.012)	0.364
	2014	0.755 [0.710;0.800]	-0.025 (0.024)	0.292	0.140 [0.103;0.177]	0.034 (0.020)	0.079	0.105 [0.074;0.137]	-0.009 (0.017)	0.602
	2015	0.726 [0.677;0.776]	**-0.064 (0.026)**	**0.015**	0.083 [0.053;0.114]	-0.016 (0.016)	0.321	0.190 [0.146;0.235]	**0.080 (0.023)**	**0.001**
	2018	0.729 [0.645;0.814]	-0.075 (0.045)	0.094	0.162 [0.088;0.236]	0.070 (0.039)	0.070	0.108 [0.051;0.165]	0.005 (0.031)	0.863
	2020	0.782 [0.723;0.842]	-0.038 (0.032)	0.223	0.104 [0.056;0.153]	0.024 (0.025)	0.344	0.113 [0.072;0.155]	0.014 (0.022)	0.514
Student	2013	0.597 [0.569;0.625]	**-0.204 (0.015)**	**<0.001**	0.205 [0.182;0.229]	**0.114 (0.012)**	**<0.001**	0.198 [0.175;0.221]	**0.089 (0.012)**	**<0.001**
	2014	0.593 [0.548;0.638]	**-0.187 (0.024)**	**<0.001**	0.226 [0.187;0.264]	**0.120 (0.020)**	**<0.001**	0.181 [0.145;0.217]	**0.067 (0.019)**	**0.001**
	2015	0.611 [0.567;0.654]	**-0.179 (0.023)**	**<0.001**	0.202 [0.166;0.238]	**0.103 (0.019)**	**<0.001**	0.187 [0.152;0.222]	**0.077 (0.019)**	**<0.001**
	2018	0.610 [0.544;0.675]	**-0.195 (0.035)**	**<0.001**	0.184 [0.133;0.236]	**0.092 (0.027)**	**0.001**	0.206 [0.153;0.260]	**0.103 (0.029)**	**<0.001**
	2020	0.698 [0.649;0.746]	**-0.123 (0.026)**	**<0.001**	0.157 [0.117;0.197]	**0.077 (0.021)**	**<0.001**	0.145 [0.109;0.181]	**0.046 (0.019)**	**0.017**

Results are based on multinomial logistic regression, with interaction (Year × EA).

Aaltonen K. *J Epidemiol Community Health* 2023; 77:160–167. doi: 10.1136/jech-2022-219706

Supplementary Table S2. Predicted probabilities (95% Confidence intervals [CI]) of reporting economic hardship including or excluding medication access problems by year and economic activity (EA), in males. Average marginal effect (AME, with standard error, se) of EA by year.

**MALE (n=59,998)**		**No hardship**			**Hardship incl. medications**			**Hardship excl. medications**		
		**Probability [95% CI]**	**AME (se)**	**p**	**Probability [95% CI]**	**AME (se)**	**p**	**Probability [95% CI]**	**AME (se)**	**p**
Full time work	2013	0.858 [0.850;0.867]			0.068 [0.062;0.074]			0.074 [0.068;0.080]		
	2014	0.845 [0.830;0.859]			0.066 [0.057;0.076]			0.089 [0.078;0.100]		
	2015	0.860 [0.846;0.873]	REF		0.062 [0.052;0.071]	REF		0.079 [0.068;0.089]	REF	
	2018	0.881 [0.863;0.899]			0.056 [0.043;0.069]			0.063 [0.049;0.077]		
	2020	0.878 [0.863;0.893]			0.054 [0.044;0.064]			0.068 [0.056;0.080]		
Part time work/retirement	2013	0.750 [0.709;0.790]	**-0.109 (0.021)**	**<0.001**	0.146 [0.112;0.180]	**0.078 (0.018)**	**<0.001**	0.104 [0.076;0.133]	**0.030 (0.015)**	**0.044**
	2014	0.749 [0.686;0.813]	**-0.095 (0.033)**	**0.004**	0.117 [0.070;0.165]	**0.051 (0.025)**	**0.040**	0.134 [0.083;0.184]	0.045 (0.026)	0.090
	2015	0.735 [0.665;0.805]	**-0.125 (0.036)**	**0.001**	0.140 [0.082;0.197]	**0.078 (0.030)**	**0.008**	0.125 [0.074;0.177]	0.047 (0.027)	0.079
	2018	0.621 [0.501;0.741]	**-0.261 (0.062)**	**<0.001**	0.288 [0.168;0.408]	**0.232 (0.062)**	**<0.001**	0.091 [0.042;0.140]	0.028 (0.026)	0.273
	2020	0.678 [0.599;0.756]	**-0.201 (0.041)**	**<0.001**	0.208 [0.141;0.275]	**0.154 (0.034)**	**<0.001**	0.114 [0.054;0.175]	0.046 (0.032)	0.142
Old age retired	2013	0.904 [0.897;0.911]	**0.046 (0.006)**	**<0.001**	0.055 [0.050;0.060]	**-0.013 (0.004)**	**0.002**	0.041 [0.036;0.045]	**-0.033 (0.004)**	**<0.001**
	2014	0.910 [0.899;0.920]	**0.065 (0.009)**	**<0.001**	0.049 [0.041;0.057]	**-0.017 (0.006)**	**0.006**	0.041 [0.034;0.049]	**-0.048 (0.007)**	**<0.001**
	2015	0.901 [0.890;0.911]	**0.041 (0.009)**	**<0.001**	0.057 [0.049;0.065]	-0.004 (0.006)	0.480	0.042 [0.035;0.049]	**-0.037 (0.006)**	**<0.001**
	2018	0.908 [0.897;0.920]	**0.027 (0.011)**	**0.014**	0.061 [0.052;0.071]	0.005 (0.008)	0.504	0.030 [0.023;0.038]	**-0.033 (0.008)**	**<0.001**
	2020	0.912 [0.903;0.922]	**0.034 (0.009)**	**<0.001**	0.048 [0.041;0.055]	-0.006 (0.006)	0.372	0.039 [0.032;0.046]	**-0.029 (0.007)**	**<0.001**
Disability/ illness	2013	0.633 [0.601;0.664]	**-0.226 (0.017)**	**<0.001**	0.265 [0.236;0.293]	**0.197 (0.015)**	**<0.001**	0.102 [0.082;0.123]	**0.028 (0.011)**	**0.008**
	2014	0.613 [0.556;0.670]	**-0.232 (0.030)**	**<0.001**	0.222 [0.174;0.270]	**0.156 (0.025)**	**<0.001**	0.165 [0.121;0.209]	**0.076 (0.023)**	**0.001**
	2015	0.617 [0.564;0.670]	**-0.242 (0.028)**	**<0.001**	0.271 [0.222;0.320]	**0.209 (0.025)**	**<0.001**	0.112 [0.077;0.147]	0.033 (0.019)	0.078
	2018	0.537 [0.453;0.621]	**-0.344 (0.044)**	**<0.001**	0.402 [0.318;0.486]	**0.346 (0.043)**	**<0.001**	0.061 [0.036;0.086]	-0.002 (0.015)	0.906
	2020	0.557 [0.491;0.623]	**-0.321 (0.035)**	**<0.001**	0.355 [0.287;0.422]	**0.301 (0.035)**	**<0.001**	0.089 [0.056;0.121]	0.020 (0.018)	0.249
Unemployed	2013	0.559 [0.527;0.591]	**-0.299 (0.017)**	**<0.001**	0.244 [0.215;0.272]	**0.176 (0.015)**	**<0.001**	0.197 [0.171;0.224]	**0.123 (0.014)**	**<0.001**
	2014	0.546 [0.493;0.599]	**-0.299 (0.028)**	**<0.001**	0.232 [0.186;0.277]	**0.165 (0.024)**	**<0.001**	0.222 [0.178;0.267]	**0.133 (0.023)**	**<0.001**
	2015	0.590 [0.542;0.639]	**-0.269 (0.026)**	**<0.001**	0.208 [0.168;0.248]	**0.146 (0.021)**	**<0.001**	0.202 [0.161;0.243]	**0.123 (0.022)**	**<0.001**
	2018	0.578 [0.505;0.652]	**-0.303 (0.039)**	**<0.001**	0.237 [0.181;0.293]	**0.181 (0.029)**	**<0.001**	0.185 [0.116;0.253]	**0.122 (0.036)**	**0.001**
	2020	0.618 [0.562;0.674]	**-0.260 (0.030)**	**<0.001**	0.195 [0.150;0.240]	**0.141 (0.024)**	**<0.001**	0.187 [0.140;0.233]	**0.119 (0.024)**	**<0.001**
Student	2013	0.683 [0.649;0.717]	**-0.175 (0.018)**	**<0.001**	0.154 [0.127;0.181]	**0.086 (0.014)**	**<0.001**	0.163 [0.136;0.190]	**0.089 (0.014)**	**<0.001**
	2014	0.663 [0.604;0.721]	**-0.182 (0.031)**	**<0.001**	0.153 [0.109;0.196]	**0.086 (0.023)**	**<0.001**	0.184 [0.136;0.233]	**0.096 (0.026)**	**<0.001**
	2015	0.716 [0.666;0.766]	**-0.144 (0.026)**	**<0.001**	0.123 [0.087;0.160]	**0.062 (0.019)**	**0.001**	0.161 [0.120;0.202]	**0.082 (0.022)**	**<0.001**
	2018	0.706 [0.617;0.795]	**-0.175 (0.046)**	**<0.001**	0.171 [0.091;0.251]	**0.115 (0.041)**	**0.005**	0.123 [0.066;0.180]	**0.060 (0.030)**	**0.043**
	2020	0.686 [0.624;0.749]	**-0.192 (0.033)**	**<0.001**	0.129 [0.082;0.176]	**0.075 (0.025)**	**0.002**	0.184 [0.133;0.236]	**0.116 (0.027)**	**<0.001**

Results are based on multinomial logistic regression, with interaction (Year × EA).
